# Latest Developments of the Julia–Kocienski Olefination Reaction: Mechanistic Considerations

**DOI:** 10.3390/molecules29122719

**Published:** 2024-06-07

**Authors:** Daniel Chrenko, Jiří Pospíšil

**Affiliations:** 1 Department of Chemical Biology, Faculty of Science, Palacky University, Šlechtitelů 27, 779 00 Olomouc, Czech Republic; daniel.chrenko01@upol.cz; 2 Department of Organic Chemistry, Faculty of Science, Palacky University, 17. Listopadu, 779 00 Olomouc, Czech Republic; 3 Laboratory of Growth Regulators, Palacky University & Institute of Experimental Botany AS CR, Šlechtitelů 27, 779 00 Olomouc, Czech Republic

**Keywords:** Julia–Kocienski reaction, olefination, reaction selectivity, reaction mechanism

## Abstract

Since its discovery, the Julia–Kocienski olefination reaction has over past 30 years become one of the key C-C connective methods that is used in late-stage natural product synthesis. The reaction proceeds under mild reaction conditions, with a wide substrate scope and functional group tolerance range and with high (*E*) selectivity. In this focused review, we discuss the reaction from a mechanistic point of view and disclose key features that play an important role in reaction selectivity. Finally, the mechanistic aspects of the newly developed modification of the Julia–Kocienski reaction, which allows the formation of both (*E*) and (*Z*) olefins from the same reaction partners, are discussed.

## 1. Introduction

Alkenes belong to a chemical functional group that is omnipresent in literally all natural products. Interestingly, since the early times when organic synthesis slowly became a ‘useful’ scientific discipline, many synthetic strategies have focused on the stereoselective synthesis of these structural motives. In particular, methods that allow for the connective stereoselective introduction of the olefin moiety have become very valuable tools for this goal. Over the past 100 years, many different connective olefination methods have been developed, although many of them follow the same retrosynthetic pathway [1]; they are based on the reunion of α-negative charge-stabilizing reagents **1** with aldehydes or ketones **2** (Table 1).

Since the introduction of the Wittig reaction [7,8] in the late 1950s of the twentieth century, the Wittig [3], Horner–Wadsworth–Emmons [4], Johnson [2], Peterson [5], and Julia olefination [1] methods have established themselves as the most widely used olefination protocols. Each of these methods has its advantages and drawbacks, which have changed over time because each of the methods has gone through a long and interesting development process since its original disclosure. In this personalized, focused review, we wish to discuss the mechanism of the so-called modified Julia reaction [1,9,10,11,12,13,14,15,16], also known as the Julia one-pot, Silvestre–Julia, or Julia–Kocienski olefination reaction, as well as its development in terms of the reaction mechanism and selectivity. The last part of the review will focus on the recently developed modification of the Julia–Kocienski olefination transformation that allows selective formation of (*E*) or (*Z*) olefins by a simple change in the reaction workup, and its scope and limitations will be compared with the Petersen and Zweifel olefination methods; protocols that also allow selective (*E*) or (*Z*) olefin formation by simple change in the reaction workup.

## 2. Origins and Mechanism of the Julia–Kocienski Olefination Reaction

### 2.1. Julia–Lythgoe Olefination vs. Julia–Kocienski Olefination: A Comparison

**Scheme 1:**
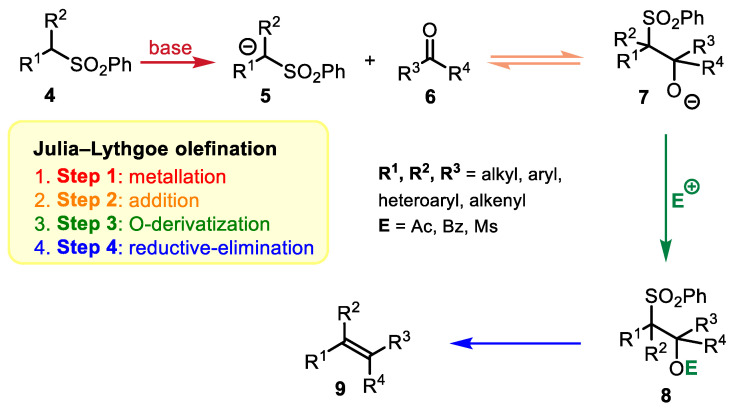
The Julia–Lythgoe olefination protocol.

Classical Julia olefination, also known as Julia–Lythgoe olefination, was described for the first time in 1973 by (Mark) Julia and Paris [17] and was later developed by Kocienski and Lythgoe [18]. The original protocol was soon expanded for the beneficial *O*-derivatization step, consisting of four distinct stages carried out commonly in the two-pot protocol (Scheme 1): (1) the metalation of an alkylarylsulfone **4**; (2) the addition of the resulting carbanion species **5** to an aldehyde or ketone **6**; (3) the *O*-acylation (sulfonylation) of the adduct **7**; (4) the elimination of the β-acyl (sulfonyl) oxysulfone **8** intermediate. The addition of **5** to **6** typically yields product **7** as a mixture of all possible diastereoisomers; however, this is not of consequence because the stereochemical information encoded in **7** (or **8**) is lost during the elimination step. A common feature of Julia–Lythgoe olefination is its high (*E*)-stereoselectivity [1]—a consequence of the various radical mechanisms that operate in the final stage of reductive elimination [19].

**Scheme 2:**
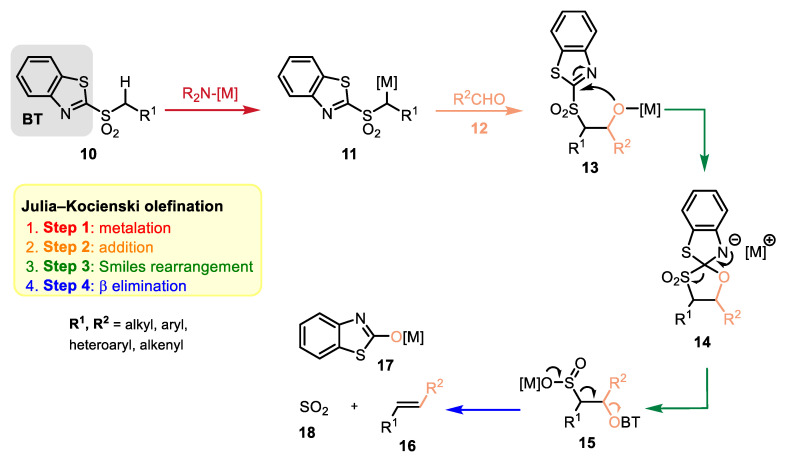
The Julia–Kocienski olefination reaction—a mechanistic overview.

The main drawbacks of Julia–Lythgoe olefination, namely the steric requirement-driven (*E*/*Z*) selectivity and the two-pot protocol, were in 1993 overcome by Silvestre Julia [20,21] (brother of Mark Julia). Their modification of the standard Julia–Lythgoe olefination protocol was based on the replacement of the phenylsulfonyl group with the benzo[*d*]thiazol-2-ylsulfonyl (BT) group (Scheme 2) [22,23,24]. The common features of the new transformation of the Julia–Lythgoe olefination reaction are the first two steps: (1) metalation; (2) the addition of metalated sulfone **11** to aldehyde **12**. Since in this case the aryl group in the alkyl aryl sulfone is an electron acceptor, the initially generated *β-*alkoxy sulfone adduct **13** can undergo a spontaneous Smiles rearrangement (S to O migration of the heteroaryl group) to yield adduct **15**. The subsequent *β*-elimination of SO_2_ (**18**) and of an aryloxide anion (**17**) in **15** directly forms olefin **16**.

**Figure 1:**
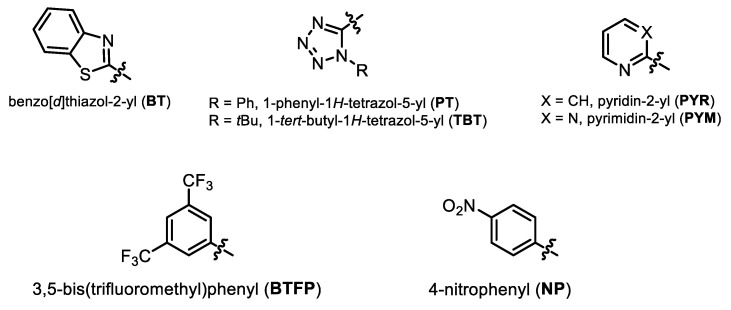
The most commonly used activators in Julia–Kocienski olefination.

As mentioned above, Silvestre Julia introduced the BT group as the only electron acceptor aryl group suitable for the Julia–Kocienski olefination reaction. However, this situation did not last long, and many other research groups introduced several different heteroaryl groups such as pyridin-2-yl (**PYR**) [20,25], 1-phenyl-1*H*-tetrazol-5-yl (**PT**) [13], 1-*tert*-butyl-1*H*-tetrazol-5-yl (**TBT**) [26], and 3,5-bis(trifluoromethyl)phenyl (**BTFP**) [20,27,28]. Interestingly, only the **PT** group introduced by Kocienski et al. [13,26] possessed sufficiently interesting properties (diminished side reactions such as homocoupling [13], high (*E*) selectivity) that remained along with the original BT group as the most widely used heteroaryl acceptor groups explored in olefination reactions (Figure 1).

The generalized scopes and limitations and the achieved (*E*/*Z*) selectivity rates observed for Julia–Lythgoe and Julia–Kocienski olefination are summarized in Table 2.

### 2.2. Reaction Mechanism and Its Impact on the Selectivity of Julia–Kocienski Olefination

**Scheme 3:**
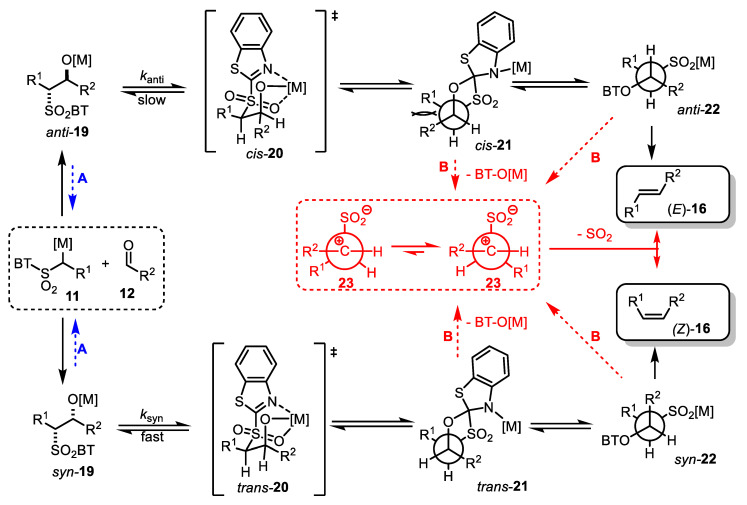
Detailed reaction mechanism of the Julia–Kocienski reaction. **A** refers to the retroaddition process that occurs for sulfones **11** with R^1^ = (hetero)aryl, alken, alkyn (see point 2 below). **B** referes to previously proposed elimination process that was based on the cation **23** formation (see point 3 below). ^‡^ indicates the transition state.

The Julia–Kocienski reaction mechanism was intensively studied by Silvestre Julia [20,21], and their studies were further extended by Kocienski and Blackmore [11,12,13,26]. Based on these excellent mechanistic studies, the reaction mechanism can be established with respect to the stereochemical outcomes of the reaction (Scheme 3). There are three key features of this mechanism that deserve a brief comment.

**Figure 2:**
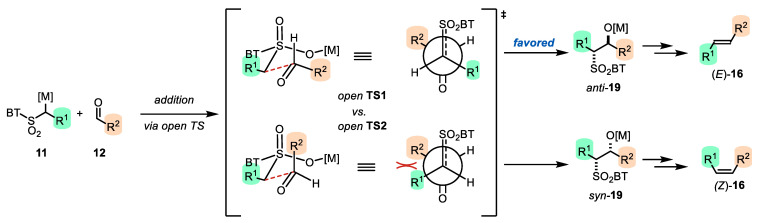
Addition of the metalated sulfone **11** to aldehyde **12**. Mechanistic rationale. ^‡^ indicates the transition state.

(1) The addition of metalated sulfone **11** to aldehyde **12** can provide *anti*-adduct *anti*-**19** via **TS1** or the *syn*-adduct *syn*-**19** via **TS2** (Figure 2). The selectivity in this step is extremely important, since all subsequent transformations of intermediate **19**, the Smiles rearrangement, and the *β*-elimination process are stereospecific. Thus, the *syn/anti*-selectivity of the addition step determines the final (*E*/*Z*) olefin ratio. Therefore, in theory, the (*E*/*Z*) selectivity of the reaction can be swapped from (*E*) to (*Z*) if proper reaction conditions are applied.
(2) When stabilized metalated sulfonyl anions **11** (R^1^ = Ph, alkenyl, etc.) are used, the addition of **11** to **12** becomes reversible (Scheme 3, path A). In this case, the original kinetically driven *syn*/*anti*-ratio of adduct **19** becomes less important in comparison with the Smiles rearrangement reaction rates (transformation of **19** to **22**). In such cases, the rearrangement of *anti*-**19** adduct leading to (*E*) olefin **16** is slower compared to the rearrangement of *syn*-**19** to olefin (*Z*)-**16** due to repulsive 1,2-interactions in the transition state (see *cis*-**20**).
(3) For the elimination step, two borderline mechanisms are generally accepted. In the first, which is the most common, the rearranged intermediate **22** undergoes *β*-elimination. The elimination is stereospecific, and the *syn*-**19** adduct-rearranged intermediate *syn*-**22** furnishes the (*Z*) olefin and the *anti*-**19** adduct-rearranged intermediate, the compound *trans*-**22** (*trans* refers to the arrangement of R^1^ and R^2^ within the intermediate cycle)**,** yields the (*E*) olefin. Alternatively, when (hetero)aryl aldehydes **12** (R^2^ = (hetero)aryl) are used, an alternative elimination pathway (path B) is postulated to occur. In this case, the elimination pathway should proceed through the formation of an intermediate carbocation **23.** The steric requirements of R^1^ and R^2^ then play a crucial role in the final (*E*/*Z*) selectivity of the reaction. Path B was used to explain the unexpected (*E*) selectivity of the coupling reactions carried out using (hetero)aryl aldehydes **12** as substrates.

**Scheme 4:**
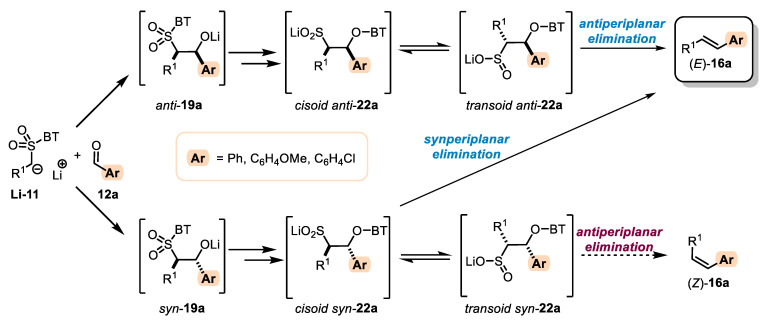
The rationale for the observed high (*E*) selectivity in the Julia–Kocienski olefination of aromatic aldehydes.

Recently, our group, in collaboration with Robiette’s group, proposed an alternative explanation for the observed (*E*) selectivity of these reactions. Our explanation is based on a combined experimental and theoretical study that revealed that the key role in the elimination step is played by the rearrangement product **22a** (Scheme 4) [14]. In general, both the *anti-* and *syn*-**22a** intermediates can adopt the *cisoid* and *transoid* conformations. The conformational equilibrium is strongly influenced by the steric requirements of the R^1^ and Ar groups, and in the case of the *anti*-**22a** intermediate, the *transoid* is preferred, while in the case of *syn-22a*, the *cisoid* is preferred. Advanced experimental and theoretical studies have suggested that in the case of a *cisoid* conformation, competitive *syn* elimination can occur [14], explaining the almost exclusive formation of (*E*) olefins observed in the general structure **16a**.

**Scheme 5:**
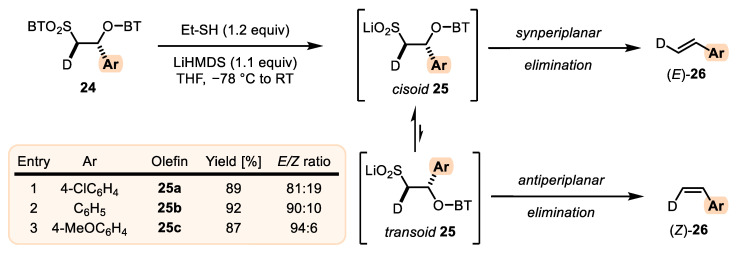
Stereoselectivity in the elimination step—a competition between the *synperiplanar* and *antiperiplanar* elimination processes.

Theoretical studies have also suggested that the *syn* elimination process should be more favored when the aryl substituent R^2^ has electron-donating substituents and disfavored when an electron-deficient substituent is present. The postulated prediction was then evaluated using a stereodefined intermediate **24**, which was selectively transformed in situ to the corresponding lithiated anion **25**, which itself was allowed to undergo an elimination process (Scheme 5). With this approach, the generated anion cannot undergo the retroaddition process (it is an intermediate after the rearrangement step), and the nucleophile generated in situ (thiolate anion) is not basic enough to trigger the epimerization process of any of the two epimerizable stereogenic centers. Therefore, only (*Z*) olefin (*Z*)-**26** should be produced as the main product of the transformation. If the reaction proceeds through the carbocation-type intermediate of **23** (see Scheme 3), an approximately 50:50 ratio of the (*E*/*Z*) isomeric mixture can be expected. In all tested cases, the (*E*)-isomer (*E*)-**26**, the product of the *synperiplanar* elimination process, was produced as the main product of the reaction, strongly suggesting that the *syn* elimination process is the main process that operates during the Julia–Kocienski olefination reaction of alkyl sulfones with aryl aldehydes. The observed stronger preference for electron-donating group-containing intermediates to undergo preferentially *synperiplanar* elimination was also in agreement with the DFT-calculation-based prediction.

### 2.3. Recent Reaction Selectivity Improvements

The reaction mechanism proposed by Julia and Kocienski, which was later confirmed by our own studies, implies that the reaction selectivity is directly linked with the initial *syn*/*anti-*selectivity of the addition step. The adduct ration further directly influences the selectivity (*E/Z*) of the overall reaction, regardless of whether the reaction proceeds through the *antiperiplanar* elimination (for R^1^ and R^2^ = alkyl) or mixed *antiperiplanar* and *synperiplanar* (for R^1^ or R^2^ = (hetero)aryl) elimination in the final step. Unsurprisingly, most of the methods developed to influence the reaction selectivity in favor of one of the two isomers focus on the key addition step.

#### 2.3.1. Solvent Effect

**Scheme 6:**
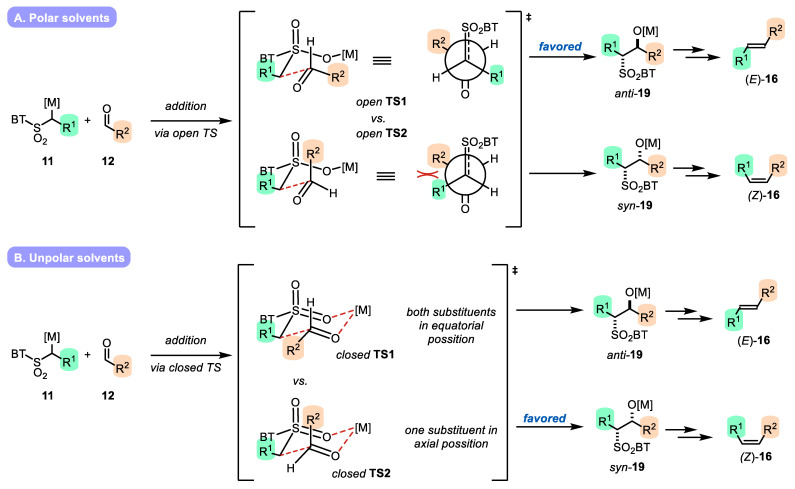
The impact of the solvent’s polarity on the stereochemical outcome of the Julia–Kocienski reaction. ^‡^ indicates the transition state.

The most important and straightforward way to influence the *syn*/*anti*-selectivity of the addition step is to choose the right solvent for the transformation. When polar solvents such as THF, DME, or DMF are used, *anti*-adduct *anti*-**19** is the preferred addition product due to its solvent stabilization potential (Scheme 6A). On the contrary, when nonpolar solvents such as toluene are used, the reaction proceeds via a closed transition state (Scheme 6B) and *syn*-adduct *syn*-**19** is preferred.

It should be noted that although such an approach is generally applicable and correct, the role of the solvent might be further influenced by metal salts and additional cosolvents.

- Metal cation

**Scheme 7:**
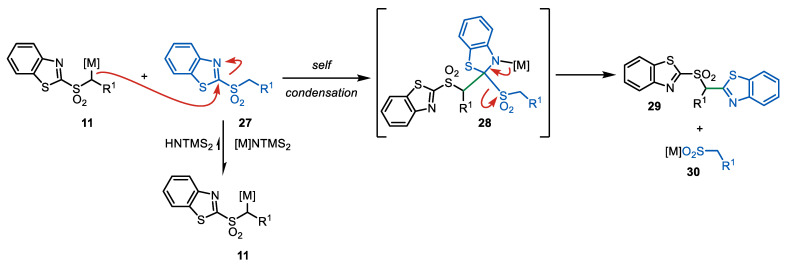
The self-condensation reaction that accompanies the reaction of anion **11**.

The metal cation, which is always present in the reaction mixture as a ‘residue’ after the deprotonation step, has a key influence on the selectivity of the reaction. In general, cations with the character of a hard Lewis acid, such as Li^+^, favor the formation of the (*E*) olefins. It is assumed that the observed (*E*) selectivity is caused by better stabilization of the generated anion **11**, which can be further added due to its lower reactivity to aldehyde **12**, which has better selectivity and favors the *anti-*adduct *anti*-**19**. On the contrary, when a large cation is used, such as K^+^, the reaction can proceed preferentially either via closed TS or the solvent can increase the dissociation of the cation from **11**, thereby increasing the reactivity. The first case is typical for nonpolar solvents (e.g., toluene) because the solvent does not provide additional stabilization to the reagents or reaction intermediates. In the latter case, the dissociation of the cation from reagent **11** increases the reactivity of the anion and leads to faster production of the kinetic product of the addition step, *anti*-isomer *anti*-**19**. However, it should also be noted that an increase in anion **11**‘s reactivity can also inevitably lead to the undesired self-condensation of reagent **11** (Scheme 7); thus, a compromise between selectivity and reactivity has to be reached.

- Cosolvents

The addition of the cosolvents to the reaction mixture can also be beneficial when (*E*) selectivity is desired. It was observed that the addition of cosolvents such as DMPU or HMPA to reaction mixtures carried out in THF or DMF led to an increase in the (*E*) olefin selectivity of the desired product. It is believed that the cosolvent’s role is in metal cation scavenging, with an impact similar to that described in the previous section (increased reactivity that favors *anti*-adduct formation). 

#### 2.3.2. Additives

Another way to increase the selectivity (*E*/*Z*) of the Julia–Kocienski reaction is by adding additives to the reaction mixture. Over the years, many different additives have been used for such purposes; however, only a few of them have had a significant effect. The relevant ones are listed below.

- Crown ethers

As mentioned in the previous section, the role of the (co-)solvent was shown to have a tremendous effect on the reaction yield and selectivity. As a modus operandi, it was postulated that polar solvents increase the reactivity of anion **11** due to a cation–anion separation (reaction kinetic) that leads to the preferential formation of *anti*-adducts (polar solvents) or *syn*-adducts (nonpolar solvents). As a disadvantage, the self-condensation of metalated sulfone **11** (Scheme 7) was observed. The use of specific cation-chelating cosolvents such as HMPA or DMPU showed only limited success, even though in several cases it led to the diminished formation of self-condensation products and an increase in (*E*) selectivity.

Based on the same logic, to increase the reactivity of metalated sulfone **11** and increase the formation of the *anti*-adduct (kinetic product), an excess of crown ethers (18-crown-6 for K^+^, 12-crown-6 for Li^+^) [29] can be used during the reaction, as demonstrated in several recent total syntheses of natural products (e.g., zeaenol [30], paecilomycins E and F [31], amphidinolide E [32], and salarins A and C [33]).

**Scheme 8:**
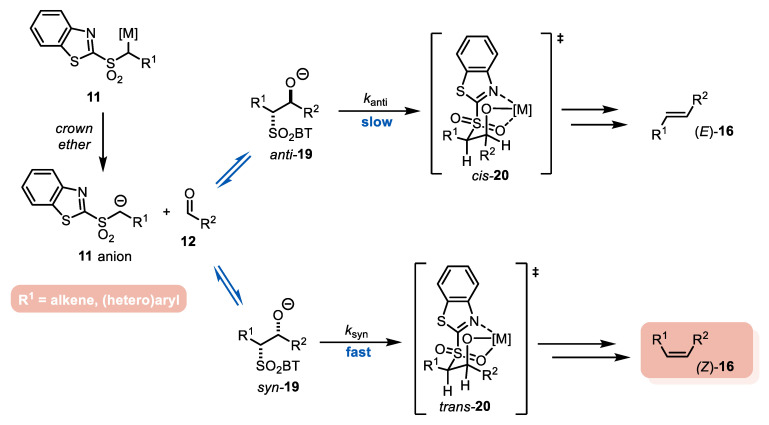
Role of crown ethers in the Julia–Kocienski reaction. High (*Z*) selectivity in the case of stabilized metalated sulfones. ^‡^ indicates the transition state.

However, it should be noted that if metalated sulfone **11** is used with a group in the lateral chain (R^1^) that is capable of stabilizing the generated anion, the addition of generated anion **11** to aldehyde **12** is reversible (Scheme 8). Consequently, the *syn*/*anti* ratio of adducts **19** is in equilibrium and (*Z*) olefin (*Z*)-**16** is formed preferentially due to a faster (*k*_anti_ < *k_syn_*) Smiles rearrangement step [34].

- Ammonium salts

The use of ammonium salts proved to also be beneficial, and in several cases of highly complex molecular scaffolds led to increases in the observed reaction yield and (*E*) selectivity [35,36]. It is believed that the role of ammonium salts is in the activation of aldehyde **12**, where due to its steric requirements it increases the *anti*-selectivity of the addition step. Note also that the role of the counter-anion of the ammonium salt is not innocent. The best (*E*) selectivity was observed when potassium-containing metalated sulfone **11** was reacted in the presence of TBAB (tetrabutylammonium bromide) and lithium-containing metalated sulfone **11** was reacted in the presence of TBAC (tetrabutylammonium chloride). Such observations suggest the beneficial formation of KBr and LiCl salts during the reaction.

- Chelating salts

Similarly, metal cations (e.g., CeCl_3_ [37,38], MgCl_2_ [39], ZnCl_2_, and LiBr) can be used to activate aldehyde **12** during the reaction. The addition of such a salt generally results in an increase in the reaction yield of the transformation. The (*E*/*Z*) selectivity of the transformation is influenced only if aldehydes bearing *α*-alkoxy substituents [39] are used in the presence of an excess of MgCl_2_ or ZnCl_2_ (addition via the Cram chelate transition state) [40].

## 3. Julia–Kocienski Olefination—Extension to Carboxylic Acid Derivatives

All of the olefination methods mentioned above are based on the reunion of the metalated sulfone **11**-type intermediate and a carbonyl-containing intermediate **12** (Scheme 2). The overall transformation can, thus, be regarded as an addition–rearrangement–elimination sequence, where the final (*E*/*Z*) selectivity of the newly olefinic bond is determined by the addition step. Therefore, the stereoselectivity is dictated by the reaction kinetic of the addition step (kinetic conditions) or by the kinetic of the rearrangement step (as the addition step is in equilibrium) (Scheme 3). Gueyrard’s group also demonstrated that in some cases lactones can also be used as reaction partners in the Julia–Kocienski reaction and that the subsequent addition–rearrangement–elimination step then yields the corresponding enol ethers [16].

**Scheme 9:**
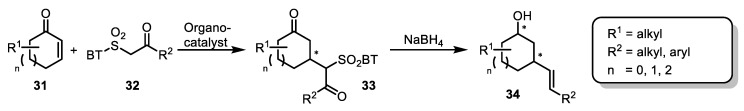
The seminal work by Jørgensen et al. [42,43] demonstrated the possibility of the stereoselective transformation of *β*-keto sulfones into the corresponding (*E*) olefins **34**. * refers to the stereogenic center.

However, recently this paradigm changed, since we introduced the ‘reaction work-up-driven selectivity’ approach for the Julia–Kocienski reaction [41]. Analogous to the famous Peterson olefination reaction [5], we designed and optimized the new Julia–Kocienski protocol, which allows selective (*E*) or (*Z*) olefin formation via a simple change in the reaction work-up procedure. Our protocol is based on the seminal work by Jørgensen et al. [42,43], which demonstrated that *β*-keto BT sulfones **33** can be successfully transformed into the corresponding olefins **34** in high yields and with (*E*) stereoselectivity (Scheme 9). 

**Scheme 10:**
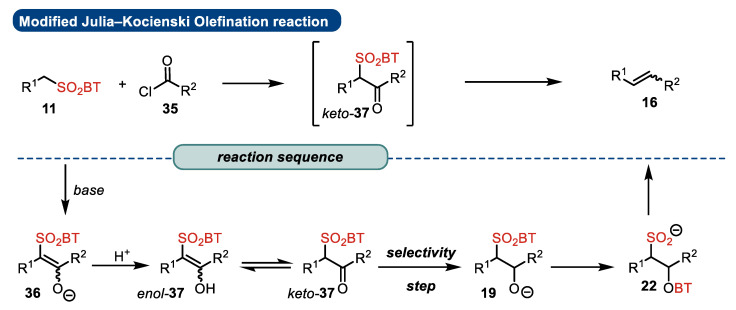
Proposed reaction sequence for the modified Julia–Kocienski olefination reaction, where the stereoselectivity of the generated olefin is not determined in the addition step.

**Scheme 11:**
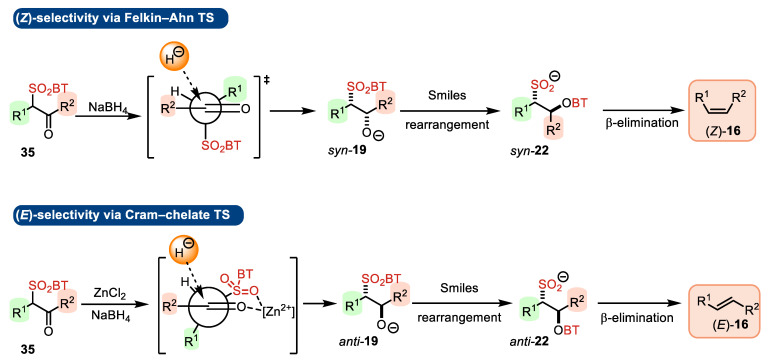
The rational design behind the stereoselective modified Julia–Kocienski olefination reaction. ^‡^ indicates the transition state.

Based on these results, we designed a novel type of Julia–Kocienski reaction that allows the synthesis of the desired olefins **16**, starting from the metalated sulfone **11** and the acyl halides **35** (Scheme 10). In this sequence, the reunion of the two reagents (compounds **11** and **35**) is carried out using a previously described protocol [44,45]. The generated adduct **36** is then quenched in situ with the external source of the proton (the protic solvent, e.g., MeOH) and the *β*-keto sulfone **37** is formed. Compound **37** is present in the reaction mixture as a dynamic mixture of its keto and enol derivatives. When an external mild reducing agent (e.g., NaBH_4_) is added, the keto form of *keto-***37** is selectively reduced, and the nucleophilic hydride approach is directed according to the Felkin–Ahn model [46] (Scheme 11). Carbonyl reduction preferentially generates a *syn* derivative of *β*-hydroxy sulfone *syn*-**19**, and compound *syn*-**19** is further converted via the Smiles rearrangement–*β*-elimination sequence of the Julia–Kocienski olefination reaction to olefin (*Z*)-**16**. However, if chelating salts such as ZnCl_2_ are added to the reaction mixture prior to NaBH_4_, the reduction proceeds through the Cram chelate model and the *anti*-*β*-hydroxy sulfone *anti*-**19** is formed. Consequently, compound *anti*-**19** then generates, after the Smiles rearrangement–*β*-elimination sequence, desired (*E*) olefin (*E*)-**16**.

Although only the preliminary scope and limitations of the transformation were established (28 examples), the method was successfully applied in the context of (nitro)fatty acid synthesis [41].

## 4. Julia–Kocienski, Peterson, and Zweifel Olefination Reactions: A Brief Comparison

**Scheme 12:**
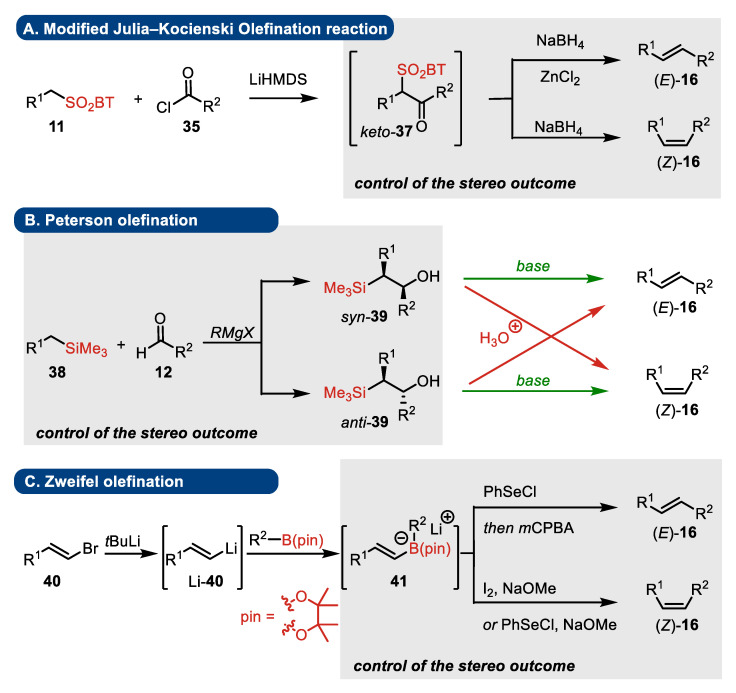
Modified Julia–Kocienski, Peterson, and Zweifel olefination reactions. Three different transition-metal-free types of connective coupling reactions that allow selective (*E*) and (*Z*) olefin formation.

In the previous chapter, we reviewed the modified Julia–Kocienski olefination reaction and disclosed its preliminary scope and limitations. In this chapter, we discuss this type of reaction in the context of the two presumably most used coupling methods that allow the generation of (*E*) or (*Z*) olefins stereoselectively during the reaction work-up—Peterson olefination [5,47] and Zweifel [48,49] olefination. Scheme 12 highlights three general schemes of the three mentioned methods. Each of the methods will now be discussed from the substrate and stereo outcome control viewpoints.

### 4.1. Modified Julia–Kocienski Reaction

- Substrates

Modified Julia–Kocienski olefination in general reunites two type substrates, sulfone **11** and acyl halide **35**. Sulfone **11** is generally obtained from the corresponding alcohol in the two-step Mitsunobu reaction [50]–oxidation protocol. Both steps generally proceed under very mild reaction conditions, since the second oxidation step is generally performed using H_2_O_2_ in the presence of molybdenum or tungsten-based catalysts [51].

- Elimination step

The mechanism of the elimination step that occurs after the decisive step controlling the stereo outcome of the reaction—the reduction of the carbonyl—was discussed in detail in the previous chapter. For the carbonyl reduction step, it should be noted that its result is strongly influenced by the steric encumbrance of the substituents on the acyl chloride **35**. Furthermore, if the R^1^ and R^2^ groups are aryl, a competitive *syn* elimination process will occur to further hammer the stereoselectivity of the reaction (see Section 2.2 for more details).

- Presence of stereogenic centers

At the present time, there are no sufficient experimental data that would experimentally address the question of the tolerance of the method toward the stereogenic center’s stability. However, one could conclude that stereogenic centers that are base- and acid-sensitive in the α position of acyl halide **35** should not be tolerated. Similarly, base-sensitive centers in and further on positions in sulfone **11** or acyl halide **35** might also undergo epimerization under the applied reaction conditions.

### 4.2. Peterson Olefination

Peterson olefination is seemingly ‘the most classical’ olefination transformation of the three methods discussed. It explores one of the ‘classical’ precursors of the olefination coupling, aldehyde, and the ratio (*E*/*Z*) of the formed olefin is determined in the first addition step [5,47]. However, there are also two characteristics that separate this type of connective method from the others: (1) the stability of the organosilicon compounds allows for further pre- or post-addition step transformations of *β*-hydroxy silanes that allow the stereoselective formation of enamines [52,53] or vinyl sulfones [54]; (2) due to the commercial availability of the TMS-CH_2_-MgCl reagent, Peterson olefination is commonly used to generate vinyl olefins from sterically hindered or perfluorinated ketones [55]. However, both trends are beyond the scope of this focused review and will not be discussed.

- Substrates

The transformation is based on the reunion of the two substrates, aldehyde **12** and silane **38** (Scheme 12). While the synthesis of the aldehyde **12** coupling partner is well documented and can be achieved via various means, under very mild reaction conditions, and on rather complex substrates, the formation of the silicon-containing partner **38** was for decades rather tricky. However, recent (past two decades) developments, especially in the field of transition-metal-mediated hydrosilylation reactions, have made available even complex silanes **38** [56,57].

As mentioned previously, the stereoselectivity outcome of the reaction is determined in the first addition step of the reaction. The addition of an anion generated from **38** to aldehyde **12** generally proceeds with reasonably good diastereoselectivity, and the influence of (co)solvents and ions is similar to those observed for the Julia–Kocienski olefination reaction (see Scheme 6). The generated adducts, anion *syn*-**42** and *anti*-**42**, then spontaneously undergo elimination via the pentacoordinate 1,2-oxasiletanide intermediate, which subsequently undergoes cycloreversion (see the elimination step below). However, when using the α-silyl organomagnesium reagent Mg-**38**, due to a strong magnesium–oxygen bond, the corresponding adduct **42** is generally sufficiently stable and can be trapped in the form of β-hydroxy silane **39** (Scheme 12B). Both generated diastereoisomers, *syn-* and *anti*-**39**, can be further separated and submitted to the stereoselective elimination step (vide infra).

- Elimination step

**Scheme 13:**
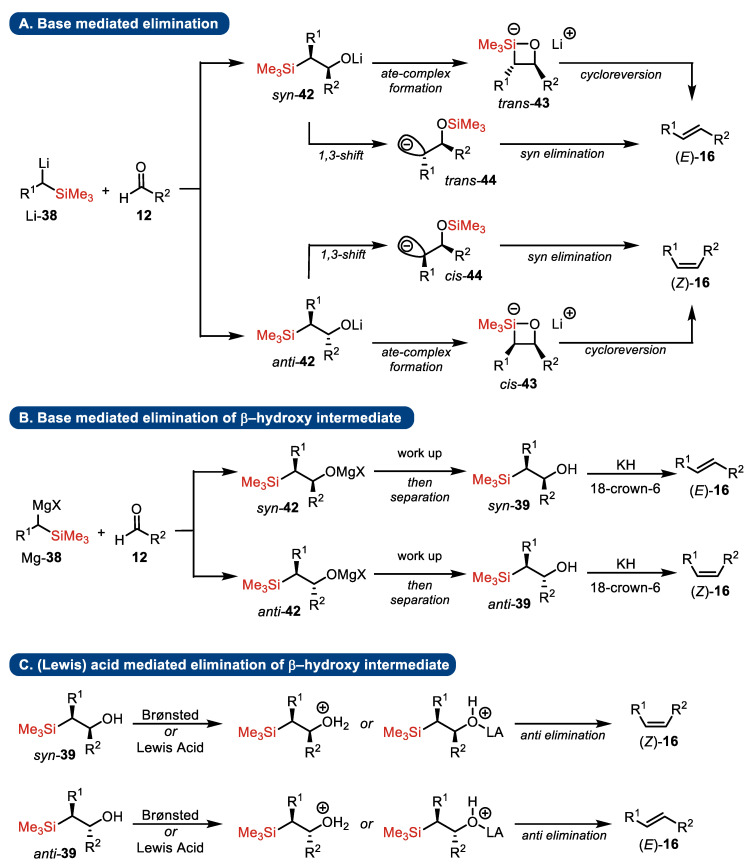
(**A**) The mechanism of Peterson olefination carried out in a one-pot manner. The mechanism of base-mediated elimination is depicted. (**B**) The Peterson olefination sequence carried out as a two-step protocol. The elimination of the *β*-hydroxy intermediate proceeds under basic conditions. (**C**) The second step of the Peterson olefination reaction, in which the elimination of the *β*-hydroxy intermediate proceeds under Brønsted or Lewis acid conditions.

As mentioned above, the elimination step in Peterson olefination generally proceeds spontaneously immediately after the addition step (Scheme 13A). In such cases, it is generally accepted that the reaction proceeds through the formation of the 1,2-oxasiletanide intermediate through the addition–cycloreversion mechanism or through the 1,3 migration–*syn*periplanar *β*-elimination mechanism. The reaction is stereoselective and the configuration of adduct **42** is reflected in the final *E*/*Z* ratio of the olefinic product **16**, since the *syn*-**42** adduct yields (*E*) olefin (*E*)-**16** and the *anti*-**42** adduct yields (*Z*) olefin (*Z*)-**16**.

Pure (*E*) or (*Z*) olefins can be obtained if interrupted Peterson olefination is performed. In such a case, the intermediate *β*-hydroxy silane **39** is isolated and the two diastereoisomers are separated. In this case, if submitted to basic conditions, the same olefin type ((*E*/*Z*) configuration) as in the one-pot protocol is obtained (Scheme 13B). However, if the intermediates *syn*- and *anti-***39** are submitted to Brønsted or Lewis acid reaction conditions, the reaction proceeds through the *anti*periplanar *β*-elimination process and the stereochemical result of the reaction is the opposite of that from base-mediated elimination. The *syn*-**39** isomer then produces (*Z*) olefin (*Z*)-**16** and the *anti*-**39** yields (*E*) olefin (*E*)-**16** (Scheme 13C).

- Presence of stereogenic centers

Similarly to Julia–Kocienski olefination, the substrates used as starting materials in the Peterson olefination reaction have stereogenic centers in the α position that are base- and acid-sensitive to the carbonyl group in **12**. Similarly, the base-sensitive centers in *β* and in positions close to the silicon group in silane **38** or aldehyde **12** could also undergo epimerization.

### 4.3. Zweifel Olefination

The Zweifel olefination protocol differs from the Julia–Kocienski and Peterson olefination reactions in many ways. Firstly, the olefinic bond found in the final product is already present in one of the two starting substrates, normally in the vinyl halide **40** (Scheme 12C). In its original form, Zweifel olefination is ‘nothing more’ than the Suzuki– Miyaura coupling-like reaction while being free of transition metals, which proceeds with the inversion of the stereochemistry when it comes to the double-bond geometry [48]. This statement is oversimplified, especially when the stereo outcome of the reaction is considered, although still states the point that the reaction is not stereodivergent, as is the case of the two previously discussed reactions. However, the situation has changed less than a decade ago, when Aggarwal and co-workers introduced a new PhSeCl-based reaction work-up protocol [58], which in combination with a base (NaOMe) or oxidant (*m*CPBA) was able to selectively produce (*E*) or (*Z*) olefins starting from the same vinyl boronic ester starting material. The difference between the newly developed reaction and the previous Zweifel olefination protocol is rather important; therefore, one could consider renaming the Zweifel stereodivergent olefination protocol as Zweifel–Aggarwal olefination.

- Substrates

The typical substrates in the Zweifel stereodivergent reaction are vinyl halide **40**, which is further transformed in situ to the corresponding lithiated species Li-**40**, and a boronic ester (normally pinacol alkyl borane). The synthesis of any of the two substrates need not to be discussed in detail, since many methods can be employed. However, what should be highlighted is that the boronic ester substrates can be readily prepared in an enantioenriched form (a stereogenic center to a boronic ester), and that the stereogenic center is due to boron migration properties conserved during the reaction (vide infra).

- Elimination step

**Scheme 14:**
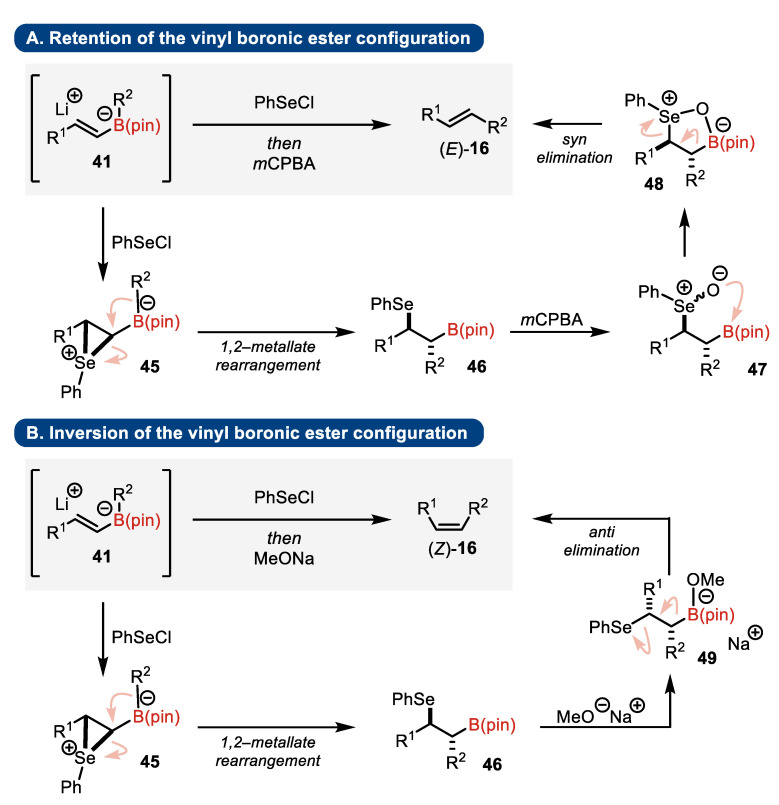
Stereodivergent Zweifel olefination reaction mechanisms (**A**) for the transformation that preserves the original vinyl boronic ester configuration and (**B**) the transformation that inverses the original vinyl boronic ester configuration.

Similarly to the previous two olefination methods, the stereodivergence of the Zweifel–Aggarwal olefination reaction is introduced in the elimination step. The common intermediate of the reaction is the borate complex **41** (shown for the (*E*) isomer), which is then reacted with the PhSeCl reagent to form intermediate **45** (Scheme 14). Intermediate **45** is highly reactive and initiates the spontaneous stereospecific 1,2-metallated migration of the alkyl group R^2^ from the boron atom. Intermediate **46** is then treated with *m*CPBA (Scheme 14A) or MeONa (Scheme 14B). In the first case, *m*CPBA oxidizes phenyl selenium to selenium oxide **47** and the generated selenium oxide **47** undergoes an intramolecular *syn* elimination process that proceeds through the cyclic intermediate **48**. In this case, the transformation proceeds with the preservation of the configuration if the original configuration of the vinyl boronate intermediate **41** is considered.

In the second case (Scheme 14B), the addition of the methanolate anion generates complex **49**. Complex **49** then spontaneously releases the phenyl selenium anion as a good leaving group via the *anti-*elimination process. In this case, a complete transformation takes place, with a formal inversion of the configuration compared to the original configuration on the vinyl boronate intermediate **41**.

Overall, starting from the same readily available 1-halide-2-alkyl/aryl olefin, both (*E*) and (*Z*) olefins **16** can be readily and stereoselectively generated.

- Presence of stereogenic centers

The Zweifel–Aggarwal olefination method has an advantage over the Julia–Kocienski and Peterson olefination methods, which results from the structure of the starting reagents; namely, it does not contain labile stereogenic centers in the α-position to the aldehyde or acyl halide. Stereogenic centers in the α-position to the boron atom or in the allylic position in the case of the second reacting partner are generally very stable under the standard reaction conditions, and in the case of boron-containing reagents, they are also readily available using various synthetic methods. From a mechanistic point of view, the 1,2-metallated rearrangement proceeds while the configuration is preserved [59], meaning this method is highly suitable for 1,2-disubstituted olefins with a stereogenic center in the allylic position. This strategy has already been exploited several times in the context of natural product synthesis [60].

## 5. Conclusions

Since its first dissemination in 1993, the reaction sequence that is now referred to as the Julia–Kocienski reaction has become a very popular late-stage connective method in natural product synthesis because it combines highly efficient (reaction yield) and selective (predominantly (*E*)-selective) connective methods that proceed in a one-pot protocol under mild reaction conditions and with broad substrate and functional group tolerances. The past 30 years of reaction development have also identified key mechanistic properties that allow for better control of the reaction selectivity. Moreover, we have recently introduced a novel modification of the Julia–Kocienski reaction that not only increases the starting material scope (since it allows for the use of previously inaccessible carboxylic acid derivatives as substrates) but also allows for selective (*E*) or (*Z*) olefin formation. In addition, this method allows for the first time the development of the Julia–Kocienski olefination reaction for the independent formation of (*E*) or (*Z*) olefins, starting from the same starting materials and using simple reaction work-up protocol alternation.

Within this focused review, we wished to shed some light on the Julia–Kocienski reaction’s development and highlight the latest evolution, which resulted in the transition of the Julia–Kocienski olefination reaction into a stereodivergent method. In this context, the modified Julia–Kocienski olefination reaction was compared with the two other methods that allow the ‘workup-based’ stereodivergent formation of (*E*) and (*Z*) olefins, the Peterson olefination and modified Zweifel olefination methods (we propose naming the latter Zweifel–Aggarwal olefination to distinguish it from the original Zweifel olefination method).

## Figures and Tables

**Table 1:**
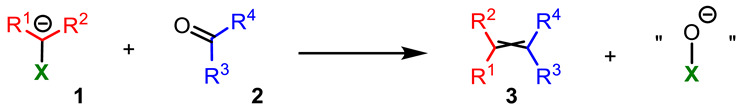
Common carbonyl-based olefination methods used in organic synthesis.

**Activating Unit X**	**Olefination Method**	**Litt. Reference**
PhSO_2_	Julia–Lythgoe	Ref. [1]
ActSO_2_	Julia–Kocienski	Ref. [1]
PhSO(NMe)	Johnson	Ref. [2]
R_3_P^+^	Wittig	Ref. [3]
R_2_P(=O)	Wittig–Horner	Ref. [3]
(RO)_2_P(=O)	Horner–Wadsworth–Emmons (HWE)	Ref. [4]
R_3_Si	Peterson	Ref. [5]
R_2_B	Boron–Wittig	Ref. [6]

**Table 2:** Comparison of the Julia–Lythgoe and Julia–Kocienski olefination reactions—general features.

Key Features	Julia–Lythgoe	Julia–Kocienski
Practical Difference	Two-pot protocol	One-pot protocol
Origin of Stereoselectivity	Reductive elimination Step	Addition step
Scope of olefin formation		
Terminal	✔	✔
1,2-disubstituted	✔	✔
Trisubstituted	✔	≈
Tetrasubstituted	≈	X
Scope of (*E*)-Stereoselectivity		
1,2-disubstituted	✔	✔
Trisubstituted	≈	X
Tetrasubstituted	≈	X
Scope of (*Z*)-Stereoselectivity		
1,2-disubstituted	X	✔ if the **TBT**-activating group is used;
Trisubstituted	X	X
Tetrasubstituted	X	X

Note: ✔—good to excellent; ≈—acceptable; X—unsatisfactory result(s).

## Data Availability

Not applicable.
